# Mixed neuroendocrine–nonneuroendocrine epithelial neoplasm of muscle invasive bladder cancer: a clinicopathologic case study

**DOI:** 10.1093/jscr/rjae612

**Published:** 2024-10-05

**Authors:** Pierre Tran, P Rama Sai, Chaya Prasad, Hanh Do, Cyrus Parsa

**Affiliations:** Department of Pathology, College of Osteopathic Medicine of the Pacific, Western University of Health Sciences, 309 E. 2nd St., Pomona, CA 91766, United States; Master of Sciences Department, University of Life Sciences, ul. Akademicka 13, 20-950 Lublin, Poland; Department of Pathology, College of Osteopathic Medicine of the Pacific, Western University of Health Sciences, 309 E. 2nd St., Pomona, CA 91766, United States; Department of Pathology, College of Osteopathic Medicine of the Pacific, Western University of Health Sciences, 309 E. 2nd St., Pomona, CA 91766, United States; Department of Pathology, College of Osteopathic Medicine of the Pacific, Western University of Health Sciences, 309 E. 2nd St., Pomona, CA 91766, United States; Department of Pathology, Beverly Hospital, Montebello, CA 90640, United States

**Keywords:** mixed neuroendocrine–nonneuroendocrine epithelial neoplasm (MiNEN), mixed bladder cancer, divergent differentiated bladder cancer, urothelial carcinoma

## Abstract

Mixed neuroendocrine–nonneuroendocrine epithelial neoplasms are rare malignant neoplasms that may occur in the bladder with highly aggressive behavior. Because of its worse prognosis, when compared to the pure urothelial carcinoma without the neuroendocrine component, the bladder mixed neuroendocrine–nonneuroendocrine epithelial neoplasm may be considered a distinct clinicopathologic entity. We present a case of mixed neuroendocrine–nonneuroendocrine epithelial neoplasm occurring in the urinary bladder of an elderly female with a personal history of chronic kidney disease, drug-resistant urinary tract infections, and neurogenic bladder. Her presenting symptoms included complaints of abdominal pain, urinary urgency, oliguria, dysuria, and occasional hematuria. Recognition of the clinicopathologic features of these rare aggressive neoplasms is important for accurate early diagnosis, necessitating appropriate therapeutic management.

## Introduction

Urothelial carcinoma has a propensity to demonstrate divergent differentiation with glandular, squamous, small-cell neuroendocrine, lymphoepithelioma-like, sarcomatoid, or other elements [1]. Neuroendocrine carcinoma (NEC) of the bladder is a very rare, highly aggressive malignancy that accounts for <1% of bladder cancers with a male prevalence in the sixth to seventh decade of life [2–4]. In ~50% of cases, they are associated with other epithelial malignancies, including a high-grade urothelial carcinoma or, more rarely, with squamous cell carcinoma or adenocarcinoma [1, 5]. In order to unify the concept of a heterogeneous group of neoplasms, epithelial malignancies occurring in a variety of organ sites that incorporate neuroendocrine neoplasms, the term ‘mixed neuroendocrine–nonneuroendocrine neoplasms (MiNENs)’ has been proposed [6]. Such MiNENs have been reported and subjected to a literature review in the pituitary, thyroid, nasal cavity, larynx, lung, digestive system, urinary system, male and female genital organs, and skin [6]. Molecular studies of such mixed neoplasms, support a monoclonal origin, possibly from a multipotential, undifferentiated stem cell present in the urothelium [1, 6, 7]. Studies suggest that urinary bladder small cell, mixed-NEC, and large cell carcinoma (LCNEC) constitute a morphological spectrum of high-grade neuroendocrine carcinoma (HGNEC) with overlapping histological features, immunophenotype, and a shared high proliferative rate [8]. In most cases, the presence of divergent morphology in a tumor with mixed histology is considered of questionable clinical importance compared with urothelial carcinomas of equal stage and grade, although some exceptions include small cell carcinoma and possibly micropapillary carcinoma [5]. Behavior of mixed tumors with neuroendocrine components is considerably more aggressive than in cases without small cell elements [1]. Because of its worse prognosis, MiNEN may be considered a distinct clinicopathologic entity in the bladder [6]. Molecular studies have highlighted the heterogeneity of these tumors with recognition of six distinct patterns [9].

**Figure 1:**
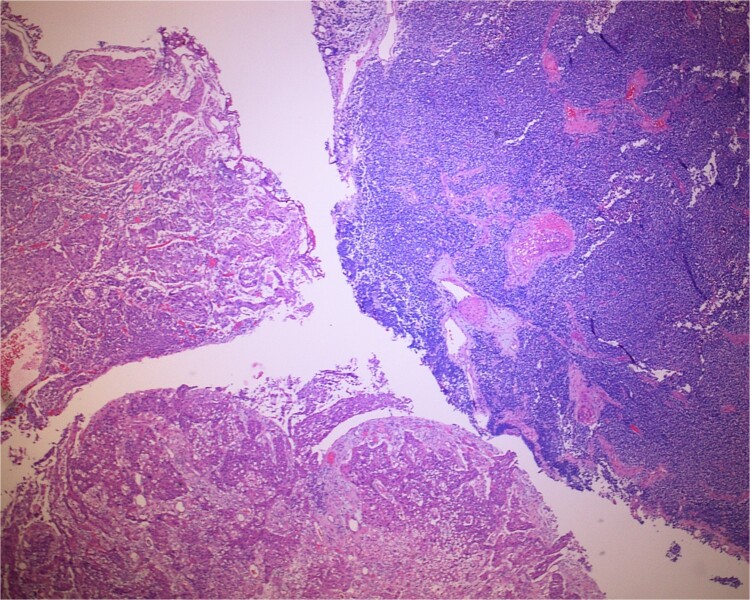
Low-power view of the neoplasm shows urothelial carcinoma on the left and neuroendocrine carcinoma on the right of the image.

**Figure 2:**
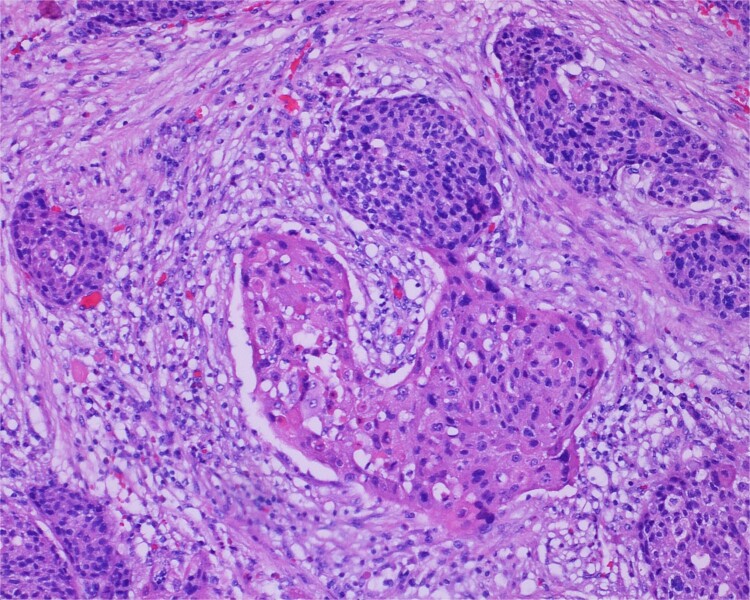
High-power view of the neoplasm shows focal merging of the high-grade neoplasm with squamoid differentiation.

In this study, we present a case of MiNEN in an 84-year-old female with longstanding urinary tract infections (UTIs) and neurogenic bladder and discuss its unique clinicopathologic features.

## Case presentation

An 84-year-old female presented to the emergency department with two days of abdominal pain, urinary urgency, oliguria, dysuria, and occasional hematuria. The patient’s personal history included chronic kidney disease, drug-resistant UTIs, neurogenic bladder, hypertension, hypercholesterolemia, and chronic neck pain. Her past surgical history included cervical fusion and total hysterectomy. The patient had a 10-year history of self-catheterization at home, a daily regimen of prophylactic doxycycline (50 mg/day), and a distant 10-year smoking history (cessation 44 years ago). The patient was seen by a urologist but did not have a cystoscopy examination for the last 10 years. She complained of an inability to urinate and was doing self-intermittent catheterization. Recently, she noticed gross hematuria. She denied fevers, chills, night sweats, and weight loss.

Her vital signs were essentially within normal limits. A physical exam was significant for bilateral lower leg pitting edema. Pertinent laboratory findings were as follows: white blood cells, 14.5 × 10^9^/L; hemoglobin, 8.8 g/dl; hematocrit, 27.8%; platelets, 800 × 10^9^/L. Urinalysis was positive for red blood cells, nitrites, and leukocytes. Her Foley catheter drained pink urine.

Renal ultrasound demonstrated severe left hydroureteronephrosis and an 8-cm heterogeneous avascular urinary bladder mass. Computed tomography (CT) of the abdomen and pelvis demonstrated mild to moderate bilateral hydroureter and hydronephrosis. Additionally, there was a 7.9-cm intraluminal, lobulated, soft tissue density within the bladder and multiple retroperitoneal lymph nodes measuring up to 1.4 cm. The cytology of the urine showed atypical cells with enlarged and irregular nuclei and an increased nuclear-cytoplasmic ratio. The patient underwent transurethral resection of the bladder mass.

**Figure 3:**
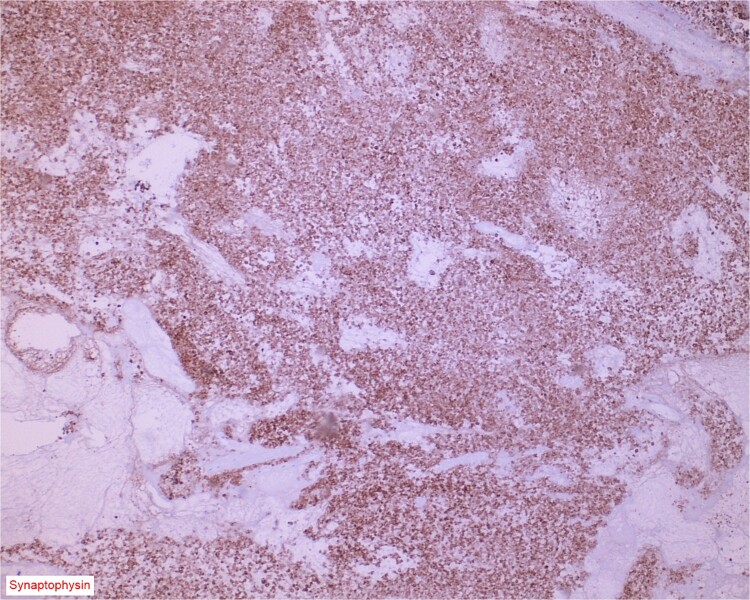
Immunostain for synaptophysin is conspicuously and diffusely positive in the neuroendocrine component of the neoplasm.

**Figure 4:**
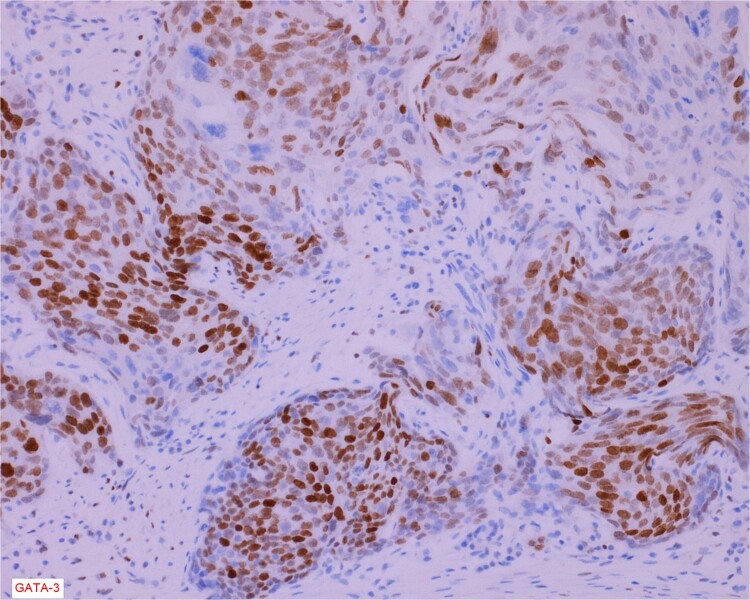
Immunostain for GATA-3 is conspicuously positive in the high grade urothelial carcinoma component.

The gross specimen consisted of multiple particles of gray friable tissue, aggregating to 58 g, 8 × 8 × 4 cm in greatest dimensions. Representative sections were submitted for histological processing and subsequent microscopic study in 12 cassettes. Histopathologic examination of the curated bladder mass revealed sheets of high-grade malignant urothelial carcinoma with adjacent areas of neuroendocrine-like differentiation (Fig. 1). There were extensive areas of tumor necrosis, extending into the muscularis propria. Merging of divergent differentiated cells was discernible in some areas (Fig. 2). Immunohistochemical stains of malignant neuroendocrine cells (Fig. 3) were positive for CD56, NSE, synaptophysin, INSM1, GATA-3 (scant), and Ki-67 (>80%), but negative for chromogranin A, TTF-1, p53, uroplakin, and Pan-CK. The malignant urothelial cells were positive for GATA-3 (Fig. 4). The histologic and IHC profiles of the curated bladder mass were consistent with invasive high-grade urothelial carcinoma, with neuroendocrine carcinoma comprising ~30% of the malignant cells.

## Discussion

The clinical and histomorphologic features presented in this paper are characteristic of bladder MiNEN with divergent cellular differentiation. Such bladder cancers are heterogeneous at the molecular level as well, and several sets of molecular classes have been proposed by a multidisciplinary expert study [9]. The recognition of the bladder cancer heterogeneity at the histopathologic and molecular level may be useful to stratify patients for prognosis or response to treatment. The molecular subtype of neuroendocrine-like class of muscle-invasive bladder cancer may share TP53 and RB1 inactivation gene mutations with the basal/squamous molecular variant [9]. The latter neoplasms with divergent cellular differentiation show poorly differentiated neuroendocrine morphology and poor survival. Variation in therapeutic options requires accurate diagnosis in order to provide optimal prognosis and survival. Nonneuroendocrine cancer may, however, play a pivotal role in the determination of life quality and prognosis even in the setting of the NEC including MiNEN [10].
